# Schwangerschaft, Geburt und Wochenbett mit SARS-CoV-2 und COVID-19 

**DOI:** 10.1007/s00129-020-04637-9

**Published:** 2020-07-13

**Authors:** C. Hagenbeck, U. Pecks, T. Fehm, F. Borgmeier, E. Schleußner, J. Zöllkau

**Affiliations:** 1 Klinik für Frauenheilkunde und Geburtshilfe, Universität Düsseldorf, Moorenstr. 5, 40225 Düsseldorf, Deutschland; 2 Campus Kiel, Klinik für Gynäkologie und Geburtshilfe, Universitätsklinikum Schleswig-Holstein, Kiel, Deutschland; 3 Klinik für Geburtsmedizin, Universität Jena, Jena, Deutschland

## Hintergrund

Mit der pandemischen Ausbreitung des neuartigen Coronavirus SARS-CoV‑2 wurden weltweit ausgedehnte Maßnahmen zur Infektionsvermeidung getroffen. Die krisenausgerichtete Anpassung des Gesundheitssystems fokussierte auf eine drastische Reduktion elektiver Diagnostik und Interventionen, um die Aufrechterhaltung kritischer Versorgungsstrukturen sicherzustellen. Da die Pränatal- und Geburtsmedizin nur sehr bedingt einem elektiven Rahmen folgen kann, nimmt die Betreuung von Schwangeren und Gebärenden mit ihren Partnern und Familien eine Sonderstellung ein. Frauenärztliche Praxen, Kliniken und Hebammenbetreuung stehen dabei vor erheblichen Herausforderungen.

Im Folgenden werden anhand der Datenlage die Besonderheiten der SARS-CoV-2 Infektion und der COVID-19 Erkrankung in Schwangerschaft, Geburt und Wochenbett mit derzeitigen Handlungsempfehlungen dargestellt (Stand Literatur 30.05.2020, CRONOS Datenbank 26.06.2020).

## Schwangerschaft

### Infektionsrisiko und Krankheitsverlauf

Derzeit gibt es keine Hinweise für ein höheres Infektionsrisiko mit SARS-CoV‑2 in der Schwangerschaft, sodass Schwangeren die allgemeinen Maßnahmen zur Infektionsvermeidung empfohlen werden. Bei einer manifesten COVID-19 Erkrankung sind die Behandlungsmöglichkeiten von Schwangeren eingeschränkt.

Insbesondere im beruflichen Umfeld muss ein erhöhtes Infektionsrisiko verhindert werden

Im Rahmen der Gefährdungsbeurteilung muss insbesondere im beruflichen Umfeld ein erhöhtes Infektionsrisiko verhindert werden. Dies betrifft besonders Arbeitsbereiche mit erhöhtem Personenkontakt. In Krankenhäusern und Arztpraxen sollten schwangere Frauen nur für patientenferne Tätigkeiten eingesetzt werden [1, 2].

Inwiefern schwangerschaftsphysiologische Veränderungen den Verlauf einer COVID-19 Erkrankung beeinflussen, ist unklar. Symptome sind bei Schwangeren in Art und Schwere vergleichbar mit denen bei Nichtschwangeren im gebärfähigen Alter [3–12]. Die Symptomhäufigkeit aus 33 Studien mit insgesamt 356 Schwangeren [13] ist in Tab. 1 dargestellt.

**Table 1:** 

Symptom	Häufigkeit (%)
Fieber	67
Husten	66
Geruchs-/Geschmacksstörungen	64
Dyspnoe	7
Halsschmerz	7
Fatigue	7
Myalgie	6
Rhinorrhö, Anorexie, Nausea/Vomitus, Kopfschmerz	<5

Schwere COVID-19 Komplikationen sind respiratorisches Versagen, Arrhythmie, akute kardiale Dekompensation und thrombembolische Ereignisse. Schwangere, die an einer COVID-19 Pneumonie erkranken, zeigen ein ähnliches Risiko intensivmedizinischer Versorgungsnotwendigkeit wie gleichaltrige Nichtschwangere [5, 6, 15]. Kritische Verläufe mit invasiver Beatmung und teilweise Notwendigkeit einer extrakorporalen Membranoxygenierung kommen vor [16]. Bei der Auswertung von über 20.000 COVID-19 Patienten konnte – im Gegensatz zu Influenzaerkrankungen – trotz ähnlicher Hospitalisierungsrate (ca. 6 %) keine erhöhte Mortalität unter Schwangeren festgestellt werden [17, 18].

### Schwangerschaftskomplikationen

Fehlgeburten treten während der SARS-CoV‑2 Pandemie nicht häufiger auf, die Datenlage ist für eine abschließende Beurteilung noch unzureichend [13, 19]. Pathogenetisch könnte dies durch die geringe trophoblastäre Expression der ACE-2-Rezeptoren erklärt werden, die den zellulären Eintrittsmechanismus des SARS-CoV‑2 darstellen. Neueste Erkenntnisse sehen eine generalisierte Endothelitis [20] mit Gerinnungsaktivierung als zentralen Pathomechanismus, der sich auch in dezidualen Arteriopathien und vermehrten intervillösen Thrombosierungen zeigte [21]. Die Hypoxämie im uteroplazentaren Strombett bei schwerer respiratorischer Insuffizienz stellt einen theoretischen Abortmechanismus dar [12, 22].

Die Frühgeburtenraten variieren je nach Studie zwischen 15 und 39 % [23, 24]. Ob diese jedoch iatrogen durch einen kritischen maternalen Zustand bedingt waren oder aber spontane Frühgeburten darstellten, ist in den Fallserien nicht ausreichend differenziert.

In den bisherigen COVID-19 Fallserien werden fetale Wachstumsrestriktionen und vermehrte intrauterine Fruchttode berichtet, eine valide Quantifizierung ist jedoch noch nicht möglich [16].

### Vertikale Transmission

Die pränatale vertikale Transmission des Virus, also eine intrauterine Übertragung von Mutter zu Kind, gilt derzeit als unwahrscheinlich [25], ähnlich wie dies für die verwandten Virenarten SARS und MERS in der Vergangenheit berichtet wurde [16]. Bislang konnte SARS-CoV‑2 weder in Fruchtwasser noch in Nabelschnurblut nachgewiesen werden. Die Virämieraten scheinen niedrig (ca. 1 %) und transient zu sein [26]. In Zusammenfassungen und Fallberichten hierzu von insgesamt 51 COVID-19 positiven Schwangeren wurde keine intrauterine Transmission festgestellt [7, 10, 12]. Jedoch wurde Ende März 2020 von 3 Neugeborenen berichtet, in deren Nabelschnurblut SARS-CoV‑2 IgM-Antikörper detektiert wurden [27, 28]. Da IgM-Antikörper eine intakte Plazentaschranke nicht passieren, lässt sich dies als Immunantwort der Feten in Folge eines intrauterinen Kontaktes mit SARS-CoV‑2 interpretieren. Plazentar wurde Virus-RNA bisher in 2 Fällen berichtet [29, 30]. Dies gelang auch bei einer febrilen COVID-19 Patientin mit Spätabort in der 20. SSW [29]. Ob dabei SARS-CoV‑2 eine kausale Rolle spielte, ist unklar, aber denkbar. Insgesamt sind zum aktuellen Zeitpunkt keine zuverlässigen Aussagen zur vertikalen Transmission zu treffen [30, 31].

Diagnostische Standards einer vertikalen prä- oder postpartalen Transmission sind derzeit nicht etabliert. Die Analyse von Nasopharynxabstrichen des Neonaten, Fruchtwasser, Amnion-Chorion-Abstrichen, Plazentagewebe (gewonnen unter aseptischen Bedingungen direkt postpartal, jeweils PCR auf virale RNA) und Nabelschnurblut (zusätzlich Testung auf SARS-CoV‑2 Antikörper) wird diskutiert. Eine zentrale Datenerfassung in Deutschland ist zu empfehlen, wie sie durch das CRONOS-Register angestrebt wird (s. unten).

### Diagnostik in der Schwangerschaft

Der Virusnachweis gelingt mit höchster Sensitivität per RT-qPCR aus geeigneten respiratorischen Materialien. Höchste bzw. hohe Nachweisraten bei klinisch klassifizierten COVID-19 Patienten zeigten die bronchoalveoläre Lavage (14 von 15 Personen; 93 %), Sputum (72 von 104; 72 %), Nasenabstriche (5 von 8; 63 %), fiberbronchoskopische Biopsie (6 von 13; 46 %), geringere fanden sich in Rachenabstrichen (126 von 398; 32 %) und Faeces (44 von 153; 29 %), kaum aber wurde SARS-CoV‑2 nachgewiesen in Blut (3 von 307; 1 %) und in Urin [26].

**Figure 1:**
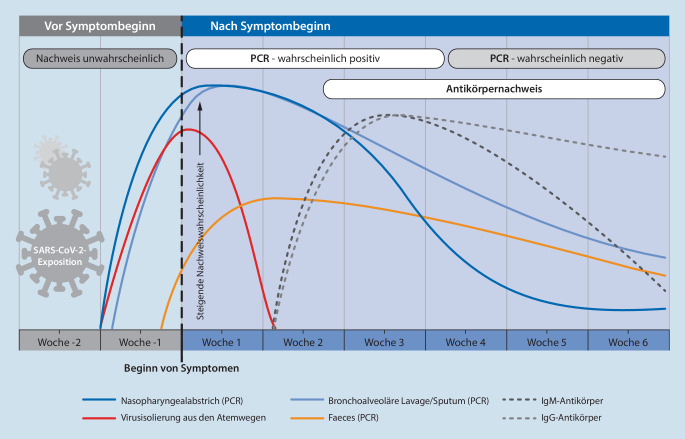

Eine veranlasste Diagnostik aufgrund respiratorischer Symptome sollte differenzialdiagnostisch weitere saisonale Erreger (u. a. Influenza, RSV) adressieren. Neben der klinischen Symptomatik und ihrem zeitlichen Verlauf muss die Dynamik von Testergebnissen (PCR oder Serologie) berücksichtigt werden ([32, 33]; Abb. 1).

Die serologische Testung virusspezifischer Antikörper (IgM, IgA, IgG) wird derzeit evaluiert [33–35]. Dieser indirekte Virusnachweis ist vor allem für Patientinnen ohne oder mit milden Symptomen und später Erstvorstellung (>2 Wochen nach Symptombeginn; PCR möglicherweise bereits negativ) von Bedeutung. ELISA-basierte Antikörpertests weisen dabei eine Spezifität von >95 % für die Diagnose von COVID-19 auf [32].

Bildgebend stellt die Computertomographie (CT) des Thorax den diagnostischen Goldstandard für die Detektion einer COVID-19 Pneumonie dar, mit einer Sensitivität von 97 % [36]. Bereits frühzeitig treten bilaterale, subpleurale milchglasartige Trübungen bis hin zu pulmonalen Konsolidierungen auf. Die sonographische Untersuchung der Lunge und Pleura kann charakteristische Veränderungen ohne mütterliche und fetale Röntgenexposition nachweisen. Die Wertigkeit der Methode wird derzeit untersucht [37–40].

### SARS-CoV-2-Prävalenz bei Schwangeren

In den Hotspot-Regionen New York City (NYC) und London wiesen systematische Screeninguntersuchungen in Entbindungskliniken einen hohen Anteil asymptomatischer infizierter Schwangerer auf. In NYC wurden im März 2020 15,4 % von 215 Gebärenden positiv auf SARS-CoV‑2 getestet, wobei nur 4 von 33 Schwangeren COVID-19 Symptome hatten – 88 % also asymptomatisch, aber infektiös zur Geburt kamen [41]. In London lag der Anteil positiv getesteter Schwangerer bei 7 % (9/129); 8 der 9 (89 %) positiven Patientinnen waren asymptomatisch [42]. In einer der Regionen mit niedrigeren COVID-19 Erkrankungsraten, in Southern Connecticut (USA), fand sich im April 2020 bei Schwangeren dagegen nur eine Prävalenz von <3 % – wiederum über 70 % von diesen waren asymptomatisch [43]. Erste deutsche Daten im gleichen Zeitraum beschreiben eine vergleichbar niedrige Periodenprävalenz von 0,6 % (95 %-KI [Konfidenzintervall] 0,01–3,1 %; [44]).

### Die asymptomatische Schwangere

#### Schwangerschaftsvorsorge

Schwangere mit Infektionsverdacht sollten sich zur Testung an die regional benannten Stellen wenden. Bei einem positiven Testergebnis ohne medizinischen Interventionsbedarf besteht keine Notwendigkeit einer unmittelbaren Vorstellung in der gynäkologischen Praxis oder Entbindungsklinik.

Wichtig ist die Aufklärung über die Befundsituation, die nach derzeitigem Kenntnisstand gute Prognose und die nicht zu erwartende vertikale Transmission. Die weitere Betreuung auch in Bezug auf eine sich manifestierende COVID-19 Erkrankung erfolgt entsprechend der Regelungen der lokalen Gesundheitsämter, die in diesen Fällen verpflichtend zu informieren sind (Infektionsschutzgesetz [IfSG]). Die Regelversorgung Schwangerer muss weiter gewährleistet sein. Mögliche psychische Belastungen [45] infolge von Quarantäne, Sorge um das Ungeborene und Geburtsfragen (z. B. Begleitung, Maskenpflicht und Besuchsregelungen) sollten frühzeitig und professionell adressiert werden [46].

#### Ultraschall und Pränataldiagnostik

Notwendige Ultraschalluntersuchungen erfolgen unabhängig einer SARS-CoV‑2 Infektion entsprechend der Mutterschaftsrichtlinien [47]. Eine häusliche Quarantäne darf hier nicht zu einer Versorgung unterhalb des Standards führen. Im Gegenteil: Darüber hinausgehende Untersuchungen erscheinen sinnvoll. Die Entwicklung einer fetalen Wachstumsrestriktion wurde in Zusammenhang mit anderen SARS-Infektionen beschrieben [48, 49]. Anhand histologischer Untersuchungen wird davon ausgegangen, dass eine Infektion mit SARS-CoV‑2 zu einer verminderten plazentaren Funktion führen kann [49, 50]. Die Fachgesellschaften empfehlen daher serielle ultrasonographische Verlaufskontrollen [51, 52] des fetalen Wachstums und der Fruchtwassermenge, sowie Dopplerkontrollen in maximal 4‑wöchentlichen Abständen.

Mögliche psychische Belastungen sollten frühzeitig professionell adressiert werden

Bei Infektion in der Frühschwangerschaft soll ein individuelles Risikoassessment erfolgen. Ein Präeklampsie-Screening erscheint vor dem Hintergrund einer möglichen plazentaren Insuffizienz infolge SARS-CoV‑2 bedingter trophoblastärer Endothelitiden sinnvoll. Die erweiterte Feindiagnostik wird von Fachgesellschaften empfohlen [52].

### Schwangere mit COVID-19

#### Ambulante Versorgung

Bei milder Symptomausprägung ist aus geburtsmedizinischer Sicht eine ambulante Betreuung möglich und sinnvoll. Die Mutterschaftsvorsorge muss unter Berücksichtigung infektionspräventiver Maßnahmen gewährleistet sein.

#### Stationäre Versorgung

Das frühe interdisziplinäre Management unter Einbeziehung von Geburtsmedizin, Neonatologie und Intensivmedizin ist bei schweren Verläufen von entscheidender Bedeutung.

Die fetale Überwachung soll neben individuellen maternal-fetalen Aspekten vor allem die fetale Versorgungssituation und Frühgeburtsbestrebungen berücksichtigen. Das maternale Monitoring der COVID-19 Pneumonie beinhaltet die periphere Sauerstoffsättigung (SpO_2_ >95 %; arterielle Blutgasanalyse >70 mm Hg PaO_2_), um eine ausreichende Oxygenierung des Feten sicherzustellen [53].

Eine spezifische antivirale Therapie ist derzeit nicht empfohlen. Vereinzelt wurden und werden schwangere COVID-19 Patientinnen mit Remdesivir, einem Nukleotidanalogon mit Aktivität gegen SARS-CoV‑2 (*in vitro*; [54]), oder anderen Wirkstoffen (Hydroxychloroquin, Chloroquin) unter Studienbedingungen behandelt. Eine antibiotische Therapie sollte einer vermuteten oder bestätigten bakteriellen Superinfektion vorbehalten bleiben und möglichst frühzeitig erreger- und resistenzgerecht erfolgen.

Es besteht ein erhöhtes Risiko für thrombembolische Ereignisse bei SARS-CoV-2 Infektion und COVID-19 Erkrankung [53]. Ursächlich hierfür scheint unter anderem eine virusbedingte Endothelitis [20]. Es besteht die Empfehlung zur medikamentösen Thromboembolieprophylaxe bei hospitalisierten Schwangeren mit positiver SARS-CoV‑2 Testung in der Anamnese [55].

#### Einsatz geburtshilflicher Medikamente

Eine antenatale Steroidgabe kann unabhängig von SARS-CoV‑2 nach geburtshilflichen Kriterien bis 34 + 0 SSW mit Betamethason bzw. Dexamethason erfolgen [17, 56]. NSAIDs können weiterhin in den Indikationen zur Präeklampsieprävention oder Wehenhemmung verwendet werden. Kardiopulmonal wirksame Medikamente wie Fenoterol sollten aufgrund ihres Nebenwirkungsprofils besonders zurückhaltend angewandt werden. Andere geburtshilflich typische Medikamente sind nach aktuellem Kenntnisstand ohne Beschränkungen einsetzbar.

## Geburt und Entbindung

### Entbindungszeitpunkt

Weder eine SARS-CoV‑2 Infektion noch die COVID-19 Erkrankung stellen eine eigenständige Entbindungsindikation dar. Besteht aus geburtshilflicher Sicht eine Entbindungsindikation bei einer SARS-CoV‑2 positiven Schwangeren, sollte eine Geburtseinleitung oder Schnittentbindung nicht verschoben werden. Dagegen ist eine Anpassung des Entbindungszeitpunktes, soweit geburtshilflich vertretbar, unter Berücksichtigung infektiologischer Gesichtspunkte (Abwarten bis negative PCR, klinischer Verlauf) anzustreben [57].

### Entbindungsmodus

Der Entbindungsmodus wird primär nach geburtshilflichen Kriterien bestimmt. Anfänglich wurden aus Krisenregionen in China und Italien überdurchschnittlich hohe Raten an Kaiserschnittentbindungen (70–96 %) berichtet [10, 13]. Inwieweit eine bereits zuvor bestehende lokale Entbindungsroutine in den Zentren der publizierten Fälle oder auch krisenbegleitende infrastrukturelle Umstände Einfluss auf den Entbindungsmodus hatten, bleibt unklar. In einer Untersuchung aus NYC zeigt sich mit 44,4 % eine deutlich niedrigere Sectioquote (8 von 18 Frauen, 14 initial asymptomatisch, 4 symptomatisch). Alle Indikationen waren geburtshilflich (fetaler Herztonabfall, Re-Sectio, Geburtsstillstand, misslungene Geburtseinleitung) und nicht durch SARS-CoV‑2 bedingt [6]. In einer US-amerikanischen Auswertung aus Southern Connecticut lag der Anteil von Kaiserschnittentbindungen SARS-CoV‑2 positiver Gebärender bei 33,3 % und wurde ebenfalls ausschließlich von geburtshilflicher Indikation bestimmt [43]. Die derzeitige Sectiorate in Deutschland bei SARS-CoV‑2 oder COVID-19 liegt laut CRONOS-Register bei 39,2 % (Stand 26.06.2020; [58]).

Daten, die Vorteile einer Schnittentbindung bei COVID-19 Erkrankung belegen, gibt es nicht [3, 8, 59, 60]. Ungeachtet dessen ist insbesondere bei schwer bis kritisch an COVID-19 Erkrankten mit unter Umständen intensivmedizinischem Versorgungsbedarf ein individuelles Vorgehen in interdisziplinärer Absprache unerlässlich [1, 3]. Auch die Frage nach der Häufigkeit einer Transmission – auf das Neugeborene zum einen, aber auch auf das Personal – in Abhängigkeit des Entbindungsmodus ist unbeantwortet. Im Falle einer geplanten Kaiserschnittentbindung ist eine SARS-CoV‑2 Testung analog zu den Empfehlungen nichtgeburtshilflicher elektiver Chirurgie durchzuführen [61]. Das Resultat soll vor der stationären Aufnahme vorliegen.

Die Empfehlung zur Spontangeburt bei SARS-CoV‑2 stellt internationalen Konsens dar [62, 63]. Dies spiegelt sich auch in den Daten aus Deutschland in der Registerstudie CRONOS (Tab. 2) wider. Wird der Spontanpartus angestrebt, so ist im Kreißsaal ein kontinuierliches Monitoring der fetalen Herzfrequenz [64] und der mütterlichen Vitalparameter mit SpO_2_ obligat [53]. Weiterhin ist auf eine ausgeglichene Flüssigkeitsbilanz zu achten, da eine Positivbilanzierung zu Lungenödemen und einer Verschlechterung der maternalen Oxygenierung führen kann [65]. Eine Amniotomie und die Überwachung der fetalen Herzfrequenz mittels Kopfschwartenelektrode kann bei entsprechender Indikation erfolgen, da bislang kein relevanter Virusnachweis im Vaginalsekret gezeigt werden konnte [7, 66]. Kontakt zu Stuhl, in dem nachweislich Virus enthalten sein kann [26, 67, 68], und damit auch eine Wassergeburt [64] sollten vermieden werden [69]. Ein Auspulsieren oder Ausstreichen der Nabelschnur scheint das Transmissionsrisiko auf das Neugeborene nicht zu erhöhen und kann daher durchgeführt werden [62, 64, 70].

**Table 2:** 

*Registrierte Zentren*	117
*Registrierte Patientinnen*	121 aus 43 Kliniken
*Gestationsalter bei SARS-CoV‑2 Nachweis*	6–42 SSW
*Wegen COVID-19 stationär aufgenommen*	21
*Schwerer mütterlicher COVID-19-Verlauf (Intubation oder eskaliert)*	7
*Frühabort, Spätabort , Totgeburt*	3, 1, 1
*Entbunden*	72
*Sectiorate*	39,2 %
*Iatrogen wegen COVID-19 entbunden insgesamt*	5
*Geburt vor 34* *+* *0 SSW * *(hiervon iatrogen aufgrund von COVID-19)*	2 (1)
*Geburt 34* *+* *0 bis 36* *+* *6 SSW * *(hiervon iatrogen aufgrund von COVID-19)*	10 (2)

*CRONOS *COVID-19 Related Obstetric and Neonatal Outcome Study in Germany*, SARS-CoV‑2* Severe Acute Respiratory Syndrome Coronavirus 2, *COVID-19* Coronavirus Disease 2019

### Entbindungsort

Jede Patientin ist während der Pandemie bei Aufnahme nach SARS-CoV‑2 Infektionsrisiken und -Symptomen zu befragen. Spezifische Screeningempfehlungen für Schwangere existieren derzeit nicht. Prävalenzabhängig ist ein Screening bei stationärer Aufnahme aufgrund des berichteten hohen Anteils asymptomatischer infizierter Schwangerer [41, 42] unter dem Aspekt der Infektionsprävention sinnvoll.

Das Bedecken von Mund und Nase ist bei Betreten der Entbindungsklinik empfohlen [62, 64]. Sub partu ist das Tragen eines MNS unabhängig vom SARS-CoV‑2 Status für das betreuende Personal bei jeder Entbindung empfohlen [71, 72]. Bei unklarem SARS-CoV‑2 Status der Gebärenden ist das Tragen eines MNS durch die Patientin zu diskutieren, bei positivem Status zu empfehlen, da die potenziell verstärkte Aerosolbildung v. a. in der aktiven Austreibungsperiode das Expositionsrisiko für das Personal erhöht. Dabei sollten jedoch der Oxygenierungsstatus der Mutter und das subjektive Wohlbefinden berücksichtigt werden [73].

Die vieldiskutierte Frage zu Begleitpersonen unter der Geburt wurde bereits früh von der DGGG adressiert [74]. Sie weist darauf hin, dass die WHO und die europäischen gynäkologischen Fachgesellschaften keine Evidenz dahingehend sehen, Partnerinnen und Partner von der Geburt auszuschließen, sofern sie nicht positiv auf SARS-CoV‑2 getestet sind oder Krankheitssymptome haben [63, 64].

Gebärende mit vermuteter oder gesicherter SARS-CoV‑2 Infektion sollten unter geeigneten Infektionsschutzmaßnahmen zur Entbindung vorgestellt und aufgenommen werden [63]. Hierzu gehören neben der prospektiven Festlegung von Zuständig- und Räumlichkeiten (mögliche Isolationsräume unter Anpassung der Raumlufttechnik [Unterdruck]; [31, 75]), die Simulation COVID-19 spezifischer Szenarien im multiprofessionellen Team und das Training im Gebrauch von Schutzkleidung. Schnittstellentätigkeiten, wie z. B. die Neugeborenenversorgung oder Verarbeitung und Entsorgung infektiösen Materials, müssen infektionsschutzgerecht vorbereitet werden. Das die Entbindung einer SARS-CoV‑2 positiven Gebärenden begleitende medizinische Personal sollte auf ein notwendiges Mindestmaß begrenzt werden, um die Anzahl der in direktem Patientenkontakt befindlichen Mitarbeitenden zu reduzieren [64, 76]. Das beteiligte neonatologische Team sollte frühzeitig und kontinuierlich informiert werden [64].

### Analgesie und Anästhesie sub partu

Eine SARS-CoV‑2 Infektion bzw. eine COVID-19 Erkrankung stellen keine Kontraindikationen zur Regionalanästhesie (Periduralkatheter/Spinalanästhesie) dar [62, 64, 76, 77]. Die bedarfsangepasste und suffiziente Schmerztherapie wird seitens der WHO betont [63], sie vermag eine kardiopulmonale Belastung zu reduzieren. Der Einsatz von Analgetika mit atemdepressivem Nebenwirkungsprofil sollte in Abwägung des mütterlichen Status erfolgen. Die Anwendung von Lachgas (N_2_O) sub partu wird aufgrund der Aerosolbildung kontrovers diskutiert [64, 76, 78].

## Wochenbett und Stillen

### Wochenbett

Die Betreuung im Wochenbett erfolgt in Abhängigkeit von Infektionsstatus und klinischem Zustand der Mutter. Bei zurückliegender Infektion (>14 Tage) und negativer PCR sind keine besonderen Maßnahmen zu treffen. Das postpartale Vorgehen bei infektiöser Mutter (asymptomatisch oder milde COVID-19 Erkrankung) ist nach ausführlicher Aufklärung in partizipativer Entscheidungsfindung individuell und interdisziplinär festzulegen [57, 78]. Inhaltlich müssen die Vorteile (Bonding, Mutter-Kind-Kontakt, Stillen etc.) gegenüber dem Übertragungsrisiko, der Erkrankungswahrscheinlichkeit des Neugeborenen und nicht zuletzt der entstehenden Nachteile bei Trennung von Mutter und Kind abgewogen werden [64, 79–82]. Die WHO befürwortet ausdrücklich unmittelbaren Mutter-Kind-Kontakt. Die Mütter sollen ermutigt werden zum Stillen unter adäquaten Hygienemaßnahmen, zu Haut-zu-Haut-Kontakt und zum Wahrnehmen von Rooming-in-Angeboten [63]. Beim Rooming-in finden die konsensbasierten Empfehlungen der DGPI Berücksichtigung: Tragen eines MNS, Abstand (1,5 m bzw. durchsichtige Trennwand), Händehygiene, Information zu Schleimhaut‑/Hautkontakt („Streicheln – ja, Küssen – nein“; [81]). Kinder von Patientinnen mit SARS-CoV‑2 Infektion sollten per Nasopharynx- und Rachenabstrich (PCR) getestet [83] und von anderen Säuglingen isoliert werden [82].

Eine Thromboembolieprophylaxe (s. oben) sollte fortgeführt werden, da wachsende Evidenz für ein erhöhtes thrombembolisches Risiko SARS-CoV‑2 positiver Patienten besteht. Diskutiert werden das Absetzen bei Entlassung [1] vs. 10–14 Tage post partum [65] bzw. die Fortführung für 6 Wochen post partum bei zusätzlichen Risikofaktoren.

Im Rahmen der der ambulanten Wochenbettbetreuung müssen pandemiebedingte Einflüsse in dieser psychisch vulnerablen Situation berücksichtigt werden [64, 84]. Ein Screeningtool zur Wochenbettdepression ist die Edinburgh Postnatal Depression Scale [85], es sollte großzügig eingesetzt werden [86].

### Stillen

Das Virus wurde in Muttermilch bislang in einem Fall in China [9] und jüngst in Deutschland nachgewiesen [87]. Weitere Analysen erbrachten keinen Virusnachweis [13]. Eine RT-qPCR aus Muttermilch sollte nach Meinung der Autoren in Erwägung gezogen werden [87].

Stillen wird auch bei maternaler SARS-CoV‑2 Infektion im internationalen Konsens befürwortet

Das Stillen wird auch bei SARS-CoV‑2 Infektion der Mutter im internationalen Konsens der Fachgesellschaften befürwortet [50, 64, 78, 81, 82, 88–90].

Neben den zahlreichen Vorteilen des Stillens ist ein möglicher passiver Immunschutz durch das Stillen denkbar, SARS-CoV‑2 spezifische Daten hierzu existieren nicht. Eine Infektion über Tröpfchen oder Aerosole durch unmittelbare Nähe zu den mütterlichen Atemwegen ist zu berücksichtigen [59, 82]. Eine praktische Anleitung der Mutter zu den speziellen Hygieneregeln und -maßnahmen beim Stillen ist obligat [91]. Hierzu zählt neben den Maßnahmen der Atemhygiene (MNS) die Hygiene von Händen, Brust und Milchpumpen [78, 88]. Das Abpumpen und anschließende Füttern durch eine gesunde Betreuungsperson ist z. B. bei räumlicher Trennung eine mögliche Alternative [79].

## Registerstudien und das CRONOS-Projekt

Zur systematischen Erfassung und Beurteilung der geburtshilflichen Auswirkungen von SARS-CoV‑2 und COVID-19 wurden weltweit Datenregister geschaffen. Neben dem PRIORITY(Pregnancy Coronavirus Outcome Registry)-Register der USA und dem britischen Register UKOSS (UK Obstetric Surveillance System) wurde in Deutschland das CRONOS-Projekt aus dem Forschungsnetzwerk der DGPM heraus ins Leben gerufen. Das Online-basierte Datenregister soll helfen, den Verlauf der COVID-19 Erkrankung, die Auswirkungen der SARS-CoV‑2 Infektion auf die Schwangerschaft, den Feten und das Neugeborene sowie mögliche vertikale Transmissionswege zu untersuchen. Eine zugehörige Biomaterialbank ist in Planung. Eingeschlossen werden können Schwangere mit positivem SARS-CoV‑2 Testbefund unabhängig vom Schwangerschaftsalter prospektiv nach entsprechender Aufklärung und Einwilligung. CRONOS ermöglicht aber auch eine retrospektive anonymisierte Eingabe, ohne Notwendigkeit der Einwilligung der Patientin. Wöchentlich wird über den aktuellen Stand informiert (Tab. 2). Unter https://www.dgpm-online.org/ finden interessierte Kliniken weitere Informationen zur Registrierung. Die erhobenen Daten sind die Grundlage zur Etablierung belastbarer Entscheidungsprozesse der Krankenhäuser und evidenzbasierter Handlungsempfehlungen in Deutschland.

## Fazit für die Praxis

- Es besteht keine erhöhte Infektionsgefahr für Schwangere. Die Verläufe einer COVID-19 Erkrankung sind nicht schwerer als die gleichaltriger Nichtschwangerer.
- Belastbare Daten zur vertikalen intrauterinen Transmission fehlen.
- Wachstumskontrollen sind alle 2–4 Wochen empfohlen, nach Infektion im ersten und frühen zweiten Trimenon zudem eine Feindiagnostik.
- Schwangere mit vermuteter oder bestätigter COVID-19 Erkrankung sollen bei Hospitalisierung eine medikamentöse Thromboseprophylaxe mit niedermolekularem Heparin erhalten.
- Weder eine SARS-CoV-2 Infektion noch die COVID-19 Erkrankung allein stellen eine Entbindungsindikation dar. Entbindungszeitpunkt und -modus werden nach geburtshilflichen Gesichtspunkten, jedoch unter Berücksichtigung infektionsspezifischer Aspekte festgelegt.
- Eine routinemäßige postpartale Trennung von Mutter und Kind ist nicht sinnvoll.
- Stillen wird unter infektionspräventiven Aspekten empfohlen.
- Die systematische Erfassung der Behandlungsdaten von Schwangeren mit SARS-CoV‑2 ist Grundlage für belastbare Entscheidungsprozesse und evidenzbasierte Handlungsempfehlungen. Hierzu wurde das zentrale CRONOS(COVID-19 Related Obstetric and Neonatal Outcome Study in Germany)-Register der DGPM (Deutsche Gesellschaft für Perinatale Medizin) etabliert.

## Abkürzungen

ACE‑2: Angiotensin-Konversionsenzym 2
COVID-19: Coronavirus Disease 2019
CRONOS: COVID-19 Related Obstetric and Neonatal Outcome Study in Germany
DGGG: Deutsche Gesellschaft für Gynäkologie und Geburtshilfe e. V.
DGPI: Deutsche Gesellschaft für Pädiatrische Infektiologie
DGPM: Deutsche Gesellschaft für Perinatale Medizin
ELISA: Enzyme-Linked Immunosorbent Assay
Ig: Immunoglobulin
MERS: Middle East Respiratory Syndrome
MNS: Mund-Nasen-Schutz
NSAID: Non-Steroidal Anti-Inflammatory Drug
PaO_2_: Sauerstoffpartialdruck
PCR: Polymerasekettenreaktion
RNA: Ribonukleinsäure
RSV: Human Respiratory Syncytial Virus
RT-qPCR: Reverse Transcription quantitative real-time Polymerase Chain Reaction
SARS: Severe Acute Respiratory Syndrome
SARS-CoV‑2: Severe Acute Respiratory Syndrome Coronavirus 2
SpO_2_: Sauerstoffsättigung (pulsoxymetrisch)
SSW: Schwangerschaftswoche
WHO: World Health Organization

## References

[1] Donders F, Lonnee-Hoffmann R, Tsiakalos A, Mendling W, Martinez de Oliveira J, Judlin P (2020). ISIDOG recommendations concerning COVID-19 and pregnancy. Diagnostics (Basel).

[2] Coronaviridae Study Group of the International Committee on Taxonomy of Viruses (2020). The species Severe acute respiratory syndrome-related coronavirus: classifying 2019-nCoV and naming it SARS-CoV-2. Nat Microbiol.

[3] Chen D, Yang H, Cao Y, Cheng W, Duan T, Fan C (2020). Expert consensus for managing pregnant women and neonates born to mothers with suspected or confirmed novel coronavirus (COVID-19) infection. Int J Gynaecol Obstet.

[4] Yang Z, Wang M, Zhu Z, Liu Y (2020). Coronavirus disease 2019 (COVID-19) and pregnancy: a systematic review. J Matern Fetal Neonatal Med.

[5] Wu Z, McGoogan JM (2020). Characteristics of and important lessons from the Coronavirus disease 2019 (COVID-19) outbreak in China: summary of a report of 72314 cases from the Chinese center for disease control and prevention. JAMA.

[6] Breslin N, Baptiste C, Gyamfi-Bannerman C, Miller R, Martinez R, Bernstein K (2020). COVID-19 infection among asymptomatic and symptomatic pregnant women: two weeks of confirmed presentations to an affiliated pair of New York City hospitals. Am J Obstet Gynecol MFM.

[7] Schwartz DA (2020). An analysis of 38 pregnant women with COVID-19, their newborn infants, and maternal-fetal transmission of SARS-coV-2: maternal coronavirus infections and pregnancy outcomes. Arch Pathol Lab Med.

[8] Khan S, Jun L, Nawsherwan, Siddique R, Li Y, Han G (2020). Association of COVID-19 with pregnancy outcomes in health-care workers and general women. Clin Microbiol Infect.

[9] Wu Y, Liu C, Dong L, Zhang C, Chen Y, Liu J (2020). Coronavirus disease 2019 among pregnant Chinese women: case series data on the safety of vaginal birth and breastfeeding. BJOG.

[10] Gatta DAN, Rizzo R, Pilu G, Simonazzi G (2020). COVID19 during pregnancy: a systematic review of reported cases. Am J Obstet Gynecol.

[11] Di Mascio D, Khalil A, Saccone G, Rizzo G, Buca D, Liberati M (2020). Outcome of Coronavirus spectrum infections (SARS, MERS, COVID 1‑19) during pregnancy: a systematic review and meta-analysis. Am J Obstet Gynecol MFM.

[12] Qiancheng X, Jian S, Lingling P, Lei H, Xiaogan J, Weihua L (2020). Coronavirus disease 2019 in pregnancy. Int J Infect Dis.

[13] Elshafeey F, Magdi R, Hindi N, Elshebiny M, Farrag N, Mahdy S (2020). A systematic scoping review of COVID-19 during pregnancy and childbirth. Int J Gynaecol Obstet.

[14] Spinato G, Fabbris C, Polesel J, Cazzador D, Borsetto D, Hopkins C (2020). Alterations in Smell or Taste in Mildly Symptomatic Outpatients With SARS-CoV-2 Infection. JAMA.

[15] Awadasseid A, Wu Y, Tanaka Y, Zhang W (2020). Initial success in the identification and management of the coronavirus disease 2019 (COVID-19) indicates human-to-human transmission in Wuhan, China. Int J Biol Sci.

[16] Mullins E, Evans D, Viner RM, O’Brien P, Morris E (2020). Coronavirus in pregnancy and delivery: rapid review. Ultrasound Obstet Gynecol.

[17] RCOG (2020). Coronavirus (COVID-19) infection in pregnancy.

[18] Docherty AB, Harrison EM, Green CA, Hardwick HE, Pius R, Norman L (2020). Features of 20 133 UK patients in hospital with covid-19 using the ISARIC WHO Clinical Characterisation Protocol: prospective observational cohort study. BMJ.

[19] Yang H, Sun G, Tang F, Peng M, Gao Y, Peng J (2020). Clinical features and outcomes of pregnant women suspected of coronavirus disease 2019. J Infect.

[20] Varga Z, Flammer AJ, Steiger P, Haberecker M, Andermatt R, Zinkernagel AS (2020). Endothelial cell infection and endotheliitis in COVID-19. Lancet.

[21] Shanes ED, Mithal LB, Otero S, Azad HA, Miller ES, Goldstein JA (2020). Placental pathology in COVID-19. Am J Clin Pathol.

[22] Lamouroux A, Attie-Bitach T, Martinovic J, Leruez-Ville M, Ville Y (2020). Evidence for and against vertical transmission for SARS-CoV‑2 (COVID-19). Am J Obstet Gynecol.

[23] Zaigham M, Andersson O (2020). Maternal and perinatal outcomes with COVID-19: a systematic review of 108 pregnancies. Acta Obstet Gynecol Scand.

[24] Dashraath P, Wong JLJ, Lim MXK, Lim LM, Li S, Biswas A (2020). Coronavirus disease 2019 (COVID-19) pandemic and pregnancy. Am J Obstet Gynecol.

[25] Juan J, Gil MM, Rong Z, Zhang Y, Yang H, Poon LC (2020). Effects of coronavirus disease 2019 (COVID-19) on maternal, perinatal and neonatal outcomes: a systematic review. Ultrasound Obstet Gynecol.

[26] Wang W, Xu Y, Gao R, Lu R, Han K, Wu G (2020). Detection of SARS-CoV‑2 in different types of clinical specimens. JAMA.

[27] Dong L, Tian J, He S, Zhu C, Wang J, Liu C (2020). Possible vertical transmission of SARS-CoV‑2 from an infected mother to her newborn. JAMA.

[28] Zeng H, Xu C, Fan J, Tang Y, Deng Q, Zhang W (2020). Antibodies in infants born to mothers with COVID-19 pneumonia. JAMA.

[29] Baud D, Greub G, Favre G, Gengler C, Jaton K, Dubruc E (2020). Second-trimester miscarriage in a pregnant woman with SARS-CoV‑2 infection. JAMA.

[30] Patane L, Morotti D, Giunta MR, Sigismondi C, Piccoli MG, Frigerio L (2020). Vertical transmission of COVID-19: SARS-CoV‑2 RNA on the fetal side of the placenta in pregnancies with COVID-19 positive mothers and neonates at birth. Am J Obstet Gynecol.

[31] Poon LC, Yang H, Dumont S, Lee JCS, Copel JA, Danneels L (2020). ISUOG interim guidance on coronavirus disease 2019 (COVID-19) during pregnancy and puerperium: information for healthcare professionals—an update. Ultrasound Obstet Gynecol.

[32] Sethuraman N, Jeremiah SS, Ryo A (2020). Interpreting diagnostic tests for SARS-CoV‑2. JAMA.

[33] Jin Y, Wang M, Zuo Z, Fan C, Ye F, Cai Z (2020). Diagnostic value and dynamic variance of serum antibody in coronavirus disease 2019. Int J Infect Dis.

[34] Hou H, Wang T, Zhang B, Luo Y, Mao L, Wang F (2020). Detection of IgM and IgG antibodies in patients with coronavirus disease 2019. Clin Transl Immunol.

[35] Zullo F, Di Mascio D, Saccone G (2020). COVID-19 antibody testing in pregnancy. Am J Obstet Gynecol.

[36] Ai T, Yang Z, Hou H, Zhan C, Chen C, Lv W (2020). Correlation of chest CT and RT-PCR testing in coronavirus disease 2019 (COVID-19) in China: a report of 1014 cases. Radiology.

[37] Buonsenso D, Moro F, Inchingolo R, Smargiassi A, Demi L, Soldati G (2020). Effectiveness of a “fast lung ultrasound teaching program” for gynecologists/obstetricians dealing with pregnant women with suspicion of COVID-19 infection. Ultrasound Obstet Gynecol.

[38] Buonsenso D, Raffaelli F, Tamburrini E, Biasucci DG, Salvi S, Smargiassi A (2020). Clinical role of lung ultrasound for the diagnosis and monitoring of COVID-19 pneumonia in pregnant women. Ultrasound Obstet Gynecol.

[39] Moro F, Buonsenso D, Moruzzi MC, Inchingolo R, Smargiassi A, Demi L (2020). How to perform lung ultrasound in pregnant women with suspected COVID-19. Ultrasound Obstet Gynecol.

[40] Kalafat E, Yaprak E, Cinar G, Varli B, Ozisik S, Uzun C (2020). Lung ultrasound and computed tomographic findings in pregnant woman with COVID-19. Ultrasound Obstet Gynecol.

[41] Sutton D, Fuchs K, D’Alton M, Goffman D (2020). Universal screening for SARS-CoV‑2 in women admitted for delivery. N. Engl J Med.

[42] Khalil A, Hill R, Ladhani S, Pattisson K, O’Brien P (2020). SARS-CoV‑2 in pregnancy: symptomatic pregnant women are only the tip of the iceberg. Am J Obstet Gynecol.

[43] Campbell KH, Tornatore JM, Lawrence KE, Illuzzi JL, Sussman LS, Lipkind HS (2020). Prevalence of SARS-CoV‑2 among patients admitted for childbirth in southern Connecticut. JAMA.

[44] Zöllkau JBM, Scherag A, Schleußner E, Groten T (2020). Periodenprävalenz von SARS-CoV‑2 in einer unselektierten Stichprobe schwangerer Frauen in Jena – Thüringen. Z Geburtshilfe. Neonatol.

[45] Wang C, Pan R, Wan X, Tan Y, Xu L, Ho CS (2020). Immediate psychological responses and associated factors during the initial stage of the 2019 coronavirus disease.

[46] RCOG (2020) Coronavirus (COVID-19) infection in pregnancy information for healthcare professionals version 9. https://www.rcog.org.uk/globalassets/documents/guidelines/2020-05-13-coronavirus-covid-19-infection-in-pregnancy.pdf (updated Wednesday 13 May 2020). Zugegriffen: 25.05.2020

[47] Kagan KO, Chaoui R (2020). Ultraschall in der Schwangerschaft wahrend der Corona-Virus Pandemie: Ein praktisches Vorgehen. Ultraschall Med..

[48] Wong SF, Chow KM, Leung TN, Ng WF, Ng TK, Shek CC (2004). Pregnancy and perinatal outcomes of women with severe acute respiratory syndrome. Am J Obstet Gynecol.

[49] Ng WF, Wong SF, Lam A, Mak YF, Yao H, Lee KC (2006). The placentas of patients with severe acute respiratory syndrome: a pathophysiological evaluation. Pathology.

[50] Stumpfe FM, Titzmann A, Schneider MO, Stelzl P, Kehl S, Fasching PA (2020). SARS-coV-2 infection in pregnancy—a review of the current literature and possible impact on maternal and neonatal outcome. Geburtshilfe Frauenheilkd.

[51] Favre G, Pomar L, Qi X, Nielsen-Saines K, Musso D, Baud D (2020). Guidelines for pregnant women with suspected SARS-CoV‑2 infection. Lancet Infect Dis.

[52] Bourne T, Leonardi M, Kyriacou C, Al-Memar M, Landolfo C, Cibula D (2020). ISUOG consensus statement on rationalization of gynecological ultrasound services in context of SARS-coV‑2. Ultrasound Obstet Gynecol.

[53] Berghella V (2020) Coronavirus disease 2019 (COVID-19): Pregnancy issues UpToDate®: UpToDate, Inc. https://www.uptodate.com/contents/coronavirus-disease-2019-covid-19-pregnancy-issues?search=covid%2019%20pregnancy&source=search_result&selectedTitle=1~150&usage_type=default&display_rank=1 ([updated May 27, 2020. Literature review current through: Apr 2020). Zugegriffen: 30.05.2020

[54] Wang M, Cao R, Zhang L, Yang X, Liu J, Xu M (2020). Remdesivir and chloroquine effectively inhibit the recently emerged novel coronavirus (2019-nCoV) in vitro. Cell Res.

[55] Thachil J, Tang N, Gando S, Falanga A, Cattaneo M, Levi M (2020). ISTH interim guidance on recognition and management of coagulopathy in COVID-19. J Thromb Haemost.

[56] ACOG (2020) COVID-19 FAQs for obstetrician-gynecologists. https://www.acog.org/clinical-information/physician-faqs/covid-19-faqs-for-ob-gyns-obstetrics. Zugegriffen: 30.05.2020

[57] Panel C‑TG (2020) Coronavirus disease 2019 (COVID-19) treatment guidelines 2020. https://files.covid19treatmentguidelines.nih.gov/guidelines/covid19treatmentguidelines.pdf. Zugegriffen: 08.05.2020

[58] Rüdiger M (2020). UP.

[59] Chen L, Li Q, Zheng D, Jiang H, Wei Y, Zou L (2020). Clinical characteristics of pregnant women with Covid-19 in Wuhan, China. N Engl J Med.

[60] Khan S, Peng L, Siddique R, Nabi G, Nawsherwan, Xue M (2020). Impact of COVID-19 infection on pregnancy outcomes and the risk of maternal-to-neonatal intrapartum transmission of COVID-19 during natural birth. Infect Control Hosp Epidemiol.

[61] DGAV e.V. (2020) COVID-19 Empfehlung der DGAV e. V. https://www.awmf.org/fileadmin/user_upload/Stellungnahmen/Medizinische_Versorgung/DGAV_COVID_Empfehlung2.pdf (updated 04/24/2020). Zugegriffen: 15.05.2020

[62] Cochrane Pregnancy and Childbirth (2020) COVID-19 review of national clinical practice guidelines for key questions relating to the care of pregnant women and their babies: Cochrane. https://pregnancy.cochrane.org/news/covid-19-review-national-clinical-practice-guidelines-key-questions-relating-care-pregnant (International Consensus). Zugegriffen: 30.05.2020

[63] WHO (2020) Q&A: pregnancy, childbirth and COVID-19. https://www.who.int/emergencies/diseases/novel-coronavirus-2019/question-and-answers-hub/q-a-detail/q-a-on-covid-19-pregnancy-and-childbirth (updated 18 March 2020). Zugegriffen: 22.05.2020

[64] Morris E, O’Brien P, Goodyear G, Relph S, Jardine J, Powell A, Gilgunn-Jones E, Mullins E, Viner R, Evans D, Ross-Davie M (2020) Coronavirus (COVID-19) infection in pregnancy—information for healthcare professionals. https://www.rcog.org.uk/globalassets/documents/guidelines/2020-05-13-coronavirus-covid-19-infection-in-pregnancy.pdf (updated 05/13/2020). Zugegriffen: 25.05.2020

[65] Stephens AJ, Barton JR, Bentum NA, Blackwell SC, Sibai BM (2020). General guidelines in the management of an obstetrical patient on the labor and delivery unit during the COVID-19 pandemic. Am J Perinatol.

[66] Qiu L, Liu X, Xiao M, Xie J, Cao W, Liu Z (2020). SARS-CoV‑2 is not detectable in the vaginal fluid of women with severe COVID-19 infection. Clin Infect Dis.

[67] Zhang W, Du RH, Li B, Zheng XS, Yang XL, Hu B (2020). Molecular and serological investigation of 2019-nCoV infected patients: implication of multiple shedding routes. Emerg Microbes Infect.

[68] Barth RE, De Regt MJA (2020). Persistence of viral RNA in stool samples from patients recovering from covid-19. BMJ.

[69] Carosso A, Cosma S, Serafini P, Benedetto C, Mahmood T (2020). How to reduce the potential risk of vertical transmission of SARS-CoV‑2 during vaginal delivery?. Eur J Obstet Gynecol Reprod Biol.

[70] Schmid MB, Fontijn J, Ochsenbein-Kolble N, Berger C, Bassler D (2020). COVID-19 in pregnant women. Lancet Infect Dis.

[71] Banala C, Moreno S, Cruz Y, Boelig RC, Saccone G, Berghella V (2020). Impact of the ACOG guideline regarding low-dose aspirin for prevention of superimposed preeclampsia in women with chronic hypertension. Am J Obstet Gynecol.

[72] CDC (2020) Clinical questions about COVID-19: questions and answers: centers for disease control and prevention. https://www.cdc.gov/coronavirus/2019-ncov/hcp/faq.html#Obstetrical-Care (updated 04/12/2020). Zugegriffen: 12.05.2020

[73] Boelig RC, Saccone G, Bellussi F, Berghella V (2020). MFM guidance for COVID-19. Am J Obstet Gynecol.

[74] DGGG e. V. (2020) DGGG Empfiehlt: Väter bei der Geburt zulassen – auch in Zeiten der Corona-Pandemie. https://www.dggg.de/presse-news/pressemitteilungen/mitteilung/dggg-empfiehlt-vaeter-bei-der-geburt-zulassen-auch-in-zeiten-der-corona-pandemie-1195/ (Pressemitteilung der DGGG). Zugegriffen: 27.05.2020

[75] Department of Health and Social Care (DHSC), Public Health Wales (PHW), Public Health Agency (PHA) Northern Ireland, Health Protection Scotland (HPS), Public Health Scotland, Public Health England, NHS England (2020) COVID-19: infection prevention and control guidance. https://assets.publishing.service.gov.uk/government/uploads/system/uploads/attachment_data/file/886668/COVID-19_Infection_prevention_and_control_guidance_complete.pdf. Zugegriffen: 28.05.2020

[76] Podovei M, Bernstein K, George R, Habib A, Kacmar R, Bateman B, Landau R (2020) Interim considerations for obstetric anesthesia care related to COVID19. https://soap.org/wp-content/uploads/2020/05/SOAP_COVID-19_Obstetric_Anesthesia_Care_052220.pdf (updated 05/22/2020). Zugegriffen: 30.05.2020

[77] Kranke P, Weibel S, Sitter M, Meybohm P, Girard T (2020). Obstetric anesthesia during the SARS-CoV-2 pandemic—a brief overview of published recommendations for action by national and international specialist societies and committees. Anasthesiol Intensivmed Notfallmed Schmerzther.

[78] GBCOG (2020) FAQ für schwangere Frauen und ihre Familien. https://www.dggg.de/fileadmin/documents/Weitere_Nachrichten/2020/20200526_GBCOG_FAQ_Corona.pdf (updated 05/26/2020). Zugegriffen: 29.05.2020

[79] CDC (2020). Interim considerations for infection prevention and control of coronavirus disease 2019 (COVID-19) in inpatient obstetric healthcare settings.

[80] Stuebe A (2020). Should infants be separated from mothers with COVID-19? First, do no harm. Breastfeed Med.

[81] DGPI (2020) Umgang mit Neugeborenen SARS-CoV‑2 positiver Mütter mit oder ohne klinische Erkrankung (COVID-19). https://dgpi.de/stellungnahme-dgpi-dggg-dgpm-umgang-mit-neugeborenen-sars-cov-2-positiver-muetter/ (updated 03/31/2020). Zugegriffen: 25.05.2020 10.1055/a-1168-2845 32503076

[82] ACOG (2020) Novel coronavirus 2019 (COVID-19). https://www.acog.org/clinical/clinical-guidance/practice-advisory/articles/2020/03/novel-coronavirus-2019 (updated 05/19/2020). Zugegriffen: 28.05.2020

[83] Puopolo KM, Hudak ML, Kimberlin DW, Cummings J (2020) Initial guidance: management of infants born to mothers with COVID-19. American Academy of Pediatrics Committee on Fetus and Newborn, Section on Neonatal Perinatal Medicine, and Committee on Infectious Diseases. https://downloads.aap.org/AAP/PDF/COVID%2019%20Initial%20Newborn%20Guidance.pdf. Zugriff: 10.05.2020

[84] Suzuki S (2020). Psychological status of postpartum women under the COVID-19 pandemic in Japan. J Matern Fetal Neonatal Med.

[85] Cox JL, Holden JM, Sagovsky R (1987). Detection of postnatal depression. Development of the 10-item Edinburgh Postnatal Depression Scale. Br J Psychiatry.

[86] Wu Y, Zhang C, Liu H, Duan C, Li C, Fan J (2020). Perinatal depressive and anxiety symptoms of pregnant women along with COVID-19 outbreak in China. Am J Obstet Gynecol.

[87] Gross R, Conzelmann C, Muller JA, Stenger S, Steinhart K, Kirchhoff F (2020). Detection of SARS-CoV‑2 in human breastmilk. Lancet.

[88] Louwen F (2020) Empfohlene Präventionsmaßnahmen für die geburtshilfliche Versorgung in deutschen Krankenhäusern und Kliniken im Zusammenhang mit dem Coronavirus: Deutsche Gesellschaft für Gynäkologie und Geburtshilfe e. V. https://www.dggg.de/fileadmin/documents/Weitere_Nachrichten/2020/COVID-19_DGGG-Empfehlungen_fuer_Kreissaele_20200319_f.pdf (updated 03/19/2020). Zugegriffen: 08.05.2020

[89] Europäisches Institut für Stillen und Laktation (2020) Coronavirus/ COVID-19 und Stillen: Aktuelle Empfehlungen. http://www.stillen-institut.com/de/coronavirus-covid-19-und-stillen-aktuelle-empfehlungen.html?fbclid=IwAR3F8_iFQDAmyHxhGHtGlXPLy8VPGKFW9DlOsFKH2cTqE2ci6_rEN7yed3c. Zugegriffen: 29.05.2020

[90] Nationale Stillkommission (2020) Stillen und COVID-19 – Stellungnahme der Nationalen Stillkommission vom 11. März 2020. https://www.mri.bund.de/de/themen/nationale-stillkommission/stellungnahmen/stillen-covid-19/ (updated 03/11/2020). Zugegriffen: 08.05.2020

[91] (2020) Breastfeeding guidance post hospital discharge for mothers or infants with suspected or confirmed SARS-CoV‑2 infection. https://services.aap.org/en/pages/2019-novel-coronavirus-covid-19-in. Zugegriffen: 08.05.2020

